# From Oral Candidiasis to Candidemia: A Review of Superficial to Invasive Progression

**DOI:** 10.1002/mbo3.70353

**Published:** 2026-07-11

**Authors:** Julia Robledo Jerez, Marcella Vieira Ambrosio, Marcela Eduarda Olegario Fernandes, Ewerton Garcia de Oliveira Mima

**Affiliations:** ^1^ Laboratory of Applied Microbiology, Department of Dental Materials and Prosthodontics, School of Dentistry São Paulo State University (UNESP) Araraquara SP Brazil

**Keywords:** antifungal resistance, *Candida* spp., candidemia, oral candidiasis, oral microbiome

## Abstract

Oral candidiasis is the most prevalent fungal infection of the oral cavity and is frequently associated with immunosuppression. Although traditionally classified as a superficial condition, growing evidence suggests that oral infection with *Candida* sp. in a susceptible host may disseminate and evolve into invasive candidiasis, such as bloodstream infection (candidemia), one of the leading nosocomial infections associated with high mortality. This review addresses the clinical manifestations of oral candidiasis, local and systemic risk factors, and the emerging role of non‐*albicans Candida* and related yeasts in the pathogenesis of both candidiasis and candidemia. Furthermore, we revisit antifungal resistance, the interaction between *Candida albicans* and the oral microbiome, and the potential impact of these interactions on progression to invasive infection. Animal models of oral candidiasis and candidemia are also considered, highlighting their relevance for understanding virulence mechanisms and for developing new therapeutic strategies. In an integrative way, this review summarizes current evidence on the relationship between oral candidiasis and candidemia, emphasizing the importance of early diagnosis, prevention, and the search for alternative therapies in light of antifungal resistance.

## Graphical abstract

Recent and limited evidence indicates that oral candidiasis can disseminate *Candida albicans* throughout the body, leading to invasive candidiasis, such as candidemia, a life‐threatening infection. Considering antifungal resistance and global outbreaks of emergent non‐*albicans Candida* and related yeasts, murine models help understand dissemination mechanisms and antifungal strategies.

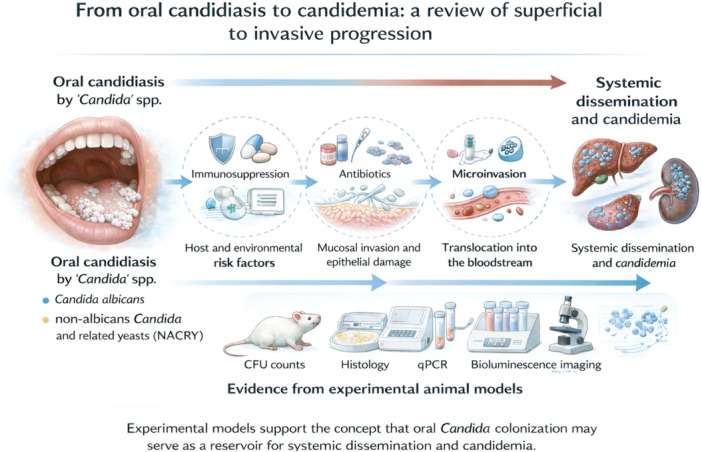

## 1 Introduction

Oral candidiasis is the main fungal infection of the oral cavity affecting newborns, the elderly, and immunosuppressed individuals. *Candida albicans* is the species most recovered from these oral lesions, but it is also found in healthy subjects. Other *Candida* species may also be isolated, such as *Candida tropicalis*, *Nakaseomyces glabratus* (former *Candida glabrata*), *Candida dubliniensis*, *Candida parapsilosis*, and *Pichia kudriavzevii* (former *Candida krusei*), but less frequently. *C. albicans* is the most prevalent and virulent species of the genus *Candida* (McManus and Coleman 2014), able to switch its morphology from yeast to filamentous form (hyphae and pseudohyphae), a process named multimorphism (or dimorphism), which is important for its virulence and potentiates its pathogenic trait (Tiwari and Dangore‐Khasbage 2024).

Although oral candidiasis is a limited superficial infection of the oral mucosa that may evolve to oropharyngeal candidiasis, its occurrence in immunosuppressed subjects raises concerns about a possible progression to systemic spread, such as invasive candidiasis, which is a deep‐seated or disseminated infection of *Candida* spp. in any organ (Lass‐Flörl et al. 2024; Pappas et al. 2018). Among invasive candidiasis, candidemia is the most severe systemic infection characterized by the presence of *Candida* in the bloodstream and one of the main nosocomial infections, especially in intensive care units (ICUs), reaching 7.7% of hospital‐acquired bloodstream infections (Tabah et al. 2023). The U.S. Centers for Disease Control and Prevention reports around 25,000 cases of candidemia in the United States each year (Centers for Disease Control and Prevention [CDC] 2024). Cases of candidemia became even more alarming with the emergence of non‐*albicans Candida* and related yeasts (NACRY), which are less susceptible to antifungal agents, resistant strains against the available drugs, and new species, such as *Candidozyma auris*, which exhibits multidrug resistance (resistant to multiple antifungals) (Lass‐Flörl et al. 2024). Currently, *C. auris* and fluconazole‐resistant *C. parapsilosis* have been associated with prolonged hospital outbreaks and represent an emerging public health concern (Daneshnia et al. 2023; Lass‐Flörl et al. 2024).

In contrast to evidence demonstrating that bacteremia may result from oral infections, such as dental caries and periodontitis (Mang‐de la Rosa et al. 2014), the direct association between oral candidiasis and candidemia is not yet well established scientifically. On the other hand, the ability of *C. albicans* to colonize the intestinal mucosa, translocate the epithelial barrier, and disseminate through the bloodstream is well established (Koh 2013; Basmaciyan et al. 2019; Sprague et al. 2022). Nonetheless, oral infection with *Candida* may serve as a fungal reservoir with the potential to disseminate systemically in immunosuppressed patients. This hypothesis is strengthened by case reports and investigations that demonstrated the presence of *Candida* in multiple anatomic sites, including the oral cavity and bloodstream (Gulati and Nobile 2016; Ramage et al. 2025). However, only one review suggests that there may be a possible association between oral candidiasis and candidemia (Ferreira et al. 2023), and currently, only a few studies in mice have evidenced such an association (Clemons et al. 2006; Mosci et al. 2014; Katagiri et al.^2018^; Kobayashi‐Sakamoto et al. 2018; Ninomiya et al. 2023; Veerapandian et al. 2025). Given *C. albicans's ability* to invade tissues, reach the bloodstream, and disseminate via the digestive system, the possibility of oral candidiasis evolving into candidemia is quite plausible, especially in immunosuppressed hosts, such as hospitalized patients. Thus, in this review, we discussed the current evidence on oral candidiasis and candidemia, based on studies involving hospitalized patients and in vivo animal models.

## 2 Oral Candidiasis

*C. albicans* is a ubiquitous commensal of the human oral microbiota isolated from healthy individuals. As an opportunistic pathogen, it can cause a range of infections, from superficial, such as oral candidiasis, to life‐threatening diseases, such as invasive candidiasis (Darwazeh et al. 2010; Talapko et al. 2021; McCarty et al. 2021). The transition from commensal to pathogen in the oral mucosa is triggered by different local factors, such as xerostomia, use of inhaled steroids, smoking, ill‐fitting dentures, and poor oral hygiene (Talapko et al. 2021). It may also be influenced by systemic factors, which include antibiotic therapy, immunosuppressive drugs, and immunocompromising diseases (HIV/AIDS, some cancers, and other conditions that impair the immune system). In addition, therapies used for cancer (chemo and radiotherapy), organ transplantation, endocrine disorders (diabetes, hypothyroidism), malnutrition (iron and vitamin deficiencies), autoimmune diseases, vulnerable age (infants and elders), and pregnancy are also risk factors for *Candida* infections (Lu 2021; Talapko et al. 2021).

This fungus is distinguished by its polymorphic nature, exhibiting four distinct classical morphological forms: yeast, hyphae, pseudohyphae, and chlamydospores (Noble et al. 2017). Yeasts are oval in shape, whereas hyphae are elongated structures that form long filaments without constrictions and with parallel walls. Pseudohyphae display characteristics of both yeasts and hyphae; in this particular form, the bud elongates and is often mistaken for true hyphae, hence the term “pseudohyphae.” However, a key distinguishing feature is that pseudohyphae have constrictions, are wider than hyphae, and tend to separate under mechanical stress (Sudbery et al. 2004). Chlamydospores are characterized by large, spherical cells with thick walls. They originate from terminal hyphal and pseudohyphal cells under conditions of hypoxia and nutrient deprivation. Although these four morphological forms are considered the most classic, *C. albicans* has also been described as exhibiting additional yeast‐like morphotypes, such as white (a) and white (α), opaque (a) and opaque (α), opaque (a/α), gray (a/α), and gastrointestinal‐induced transition cells (Noble et al. 2017). These variations depend on the colonized site and the health or disease state of the host, highlighting the remarkable plasticity of this species. Further information on this topic can be found in Noble et al. (2017).

Regardless of its morphological form, *C. albicans* possesses a wide range of attributes that favor pathogenesis. However, many studies indicate that the hyphal form is the main factor driving tissue invasion and dissemination (Gow et al. 2002; Sudbery et al. 2004). In its hyphal form, *C. albicans* can invade host tissues by endocytosis or active penetration, disrupting epithelial junctions and reaching the bloodstream, leading to systemic infection (candidemia) (Gow et al. 2002; Sudbery et al. 2004). Candidiasis can manifest in different forms, depending on the extent of the infection and the host's immune response. The most common fungal infection of the oral cavity is oral candidiasis, characterized by a white pseudomembrane that can be removed, revealing an erythematous mucosa. The infection is more prevalent in high‐risk groups, such as immunocompromised individuals and neonates, whose immune systems are not yet fully developed, and it is usually transmitted by the mother or by healthcare professionals. In addition, in the 1980s, oral candidiasis became one of the first clinical signs of HIV infection, increasing its incidence among immunocompromised patients. However, with the advancement of antiretroviral therapy (HAART), there has been a significant reduction in cases among people living with HIV (Elangovan et al. 2012; Telles et al. 2017).

Poor denture hygiene is a local etiological factor for the development of oral candidiasis, known as denture stomatitis. Studies highlight the importance of mechanical methods (brushing) and chemical methods (cleansing solutions) in the daily routine of these patients. The habit of sleeping with dentures is also a contributing factor to denture stomatitis (Gendreau and Loewy 2011).

The literature describes four categories of primary oral candidiasis: pseudomembranous candidiasis, acute erythematous candidiasis, chronic erythematous candidiasis (CEC), and chronic hyperplastic candidiasis (CHC). Each of these categories of *Candida* infection is associated with characteristic clinical signs and symptoms and with a range of host predisposing factors.

### 2.1 Acute Manifestations of Oral Candidiasis

#### 2.1.1 Pseudomembranous Candidiasis

Pseudomembranous candidiasis, popularly known as oral thrush, is a common clinical form of *Candida* infection, characterized by white plaques on the oral mucosa, frequently located on the tongue, soft palate, buccal mucosa, and lips. These lesions have a curd‐like appearance and can be easily removed with a spatula or gauze, revealing an erythematous, sometimes erosive base.

This type of candidiasis is frequently associated with corticosteroid use, particularly inhalational corticosteroids, and is also more commonly observed in neonates and immunosuppressed individuals. Diseases, such as leukemia and HIV infection, are recognized for favoring the onset of this condition, rendering pseudomembranous candidiasis an initial and, in some cases, recurrent manifestation in these populations. In immunocompromised patients, the infection can become persistent for months or even years, especially in the absence of appropriate intervention (L. P. Samaranayake et al. 2009; Lewis and Williams 2017).

#### 2.1.2 Acute Erythematous Candidiasis

Acute erythematous candidiasis, long referred to as “antibiotic sore mouth,” received this designation because its development requires a reduction in the bacterial component of the oral microbiota, which occurs after the use of broad‐spectrum antibiotics. The continuous use of steroids, particularly in inhaled form, may be an additional factor, as it can lead to local immunosuppression with consequent overgrowth of *Candida*. It may also arise from persistent acute pseudomembranous candidiasis.

Acute erythematous candidiasis presents as an erythematous lesion on the dorsum of the tongue (usually in the mid‐posterior region), the palate, or the buccal mucosa. Clinically, the presentation may be asymptomatic, requiring attention during the intraoral examination. However, in some cases, especially after antibiotic administration, such as tetracyclines, patients may report a burning sensation in the mouth. In these cases, the most evident appearance occurs on the dorsum of the tongue, with loss of filiform papillae and an erythematous appearance (L. P. Samaranayake et al. 2009; Lewis and Williams 2017).

### 2.2 Chronic Manifestations of Oral Candidiasis

#### 2.2.1 Chronic Erythematous Candidiasis

CEC is the most prevalent form of oral candidiasis and is known as denture stomatitis. Approximately, up to 75% of denture wearers present this condition, with some studies reporting a higher incidence in women (Zissis et al. 2006), although other investigations have demonstrated no association between the lesion and the gender of the patients involved (Gendreau and Loewy 2011).

The inflammation is characterized by redness of the mucosa located beneath the denture base. It is a commonly observed condition, especially in the palatal region, being less frequent in the mandible, and it is classified into three categories according to the Newton classification (1962): Grade I, corresponding to localized inflammation with hyperemic spots; Grade II, characterized by diffuse inflammation of the palate in contact with the denture; and Grade III, palatal mucosal hyperplasia (Gendreau and Loewy 2011). This alteration may occur in users of any type of acrylic denture or intraoral device.

Among the main factors that favor the development of this condition are poor oral hygiene, prolonged use of the denture without removal, and inadequate adaptation of the prosthetic device to the mucosa (Lewis and Williams 2017). The microenvironment formed between the denture and the palate, with reduced salivary and oxygen flow, promotes the development of anaerobic/facultative bacteria and *Candida* spp. (Gleiznys et al. 2015; Sakima et al. 2022). The properties of the acrylic resin of the denture, such as roughness, hydrophobicity, electrostatic interactions, and salivary pellicle formation, favor the development of *Candida* spp. biofilm (Gleiznys et al. 2015). *Candida* spp. shows greater affinity for the denture surface than for the palatal fibromucosa (Vila, Montelongo‐Jauregui, et al. 2020), the latter exhibits a self‐renewal capacity through desquamation of its outer layers, which contributes to the control of biofilm formation (Gleiznys et al. 2015).

#### 2.2.2 Chronic Hyperplastic Candidiasis (CHC)

CHC is clinically divided into two forms, known as the homogeneous form and the nodular or speckled form. The homogeneous form usually appears as a thick white plaque, while the nodular or speckled form appears as multiple white nodules distributed over an erythematous mucosal surface (Lewis and Williams 2017; Lorenzo‐Pouso et al. 2022).

It usually affects the region of the labial commissures, the dorsum of the tongue, or even the palate of denture wearers. These lesions are adherent to the mucosa and do not disappear when rubbed with gauze or scraped off. One of the concerns related to CHC is the possible association between the presence of *Candida* and malignant changes in the affected tissues (Lorenzo‐Pouso et al. 2022), although it is still unclear whether the fungus actually contributes to the development of dysplasia (McCullough et al. 2002). Therefore, some professionals recommend initiating a 7‐day course of systemic antifungal therapy before performing a biopsy of the suspected lesion (Lorenzo‐Pouso et al. 2022). In this way, if epithelial alterations are observed, it will be easier to determine whether they were truly dysplastic or only related to the fungal infection. Patients with this type of lesion often have the habit of smoking (Lewis and Williams 2017). Other predisposing factors include anemia, immunosuppression, and vitamin deficiency (Lorenzo‐Pouso et al. 2022).

In addition to primary candidiasis, secondary forms have also been reported as a consequence of systemic alterations (L. P. Samaranayake et al. 2009; Lewis and Williams 2017; Talapko et al. 2021). However, there is no consensus on the classification of secondary candidiasis, since some authors describe lesions associated with *Candida*, such as angular cheilitis and median rhomboid glossitis, as primary candidiasis (L. P. Samaranayake et al. 2009) or as secondary forms (Lewis and Williams 2017; Talapko et al. 2021). Only chronic mucocutaneous candidiasis has been established as a secondary type.

Angular cheilitis is characterized as inflammatory lesions at the corners of the mouth with erythema, fissures, and crusts that may be covered by white plaques. It is associated with vitamin and iron deficiencies, reduced vertical dimension in individuals due to tooth loss, and denture stomatitis. In addition to the presence of *Candida*, the lesions also harbor bacteria, such as *Staphylococcus aureus* and *Streptococcus* species (L. P. Samaranayake et al. 2009; Coronado‐Castellote and Jiménez‐Soriano 2013; Lewis and Williams 2017).

Median rhomboid glossitis is an asymptomatic lesion that manifests along the midline of the tongue, with a rhomboid or elliptical shape in a symmetrical pattern, involving the dorsum of the tongue anterior to the circumvallate papilla. It may present as atrophic or macular, hyperplastic or exophytic, and fissured or lobular (L. P. Samaranayake et al. 2009; Coronado‐Castellote and Jiménez‐Soriano 2013; Lewis and Williams 2017; Talapko et al. 2021). Smoking and inhaled corticosteroids are considered predisposing factors (Lewis and Williams 2017).

The fact that these lesions are often painless leads to underdiagnosis, and thus, there is a possibility of progression to a chronic disease and secondary mucocutaneous manifestations. Chronic candidiasis is classified as a form of secondary oral candidiasis. A representative example is chronic mucocutaneous candidiasis, which manifests as lesions in the oral cavity and other areas of the body. Secondary candidiasis results from diseases or treatments that reduce immunity, allowing the abnormal proliferation of the fungus (L. P. Samaranayake et al. 2009). The clinical types of oral candidiasis are summarized in Table 1.

**Table 1:** Clinical types of oral candidiasis and their main predisposing factors.

Type of injury	Clinical description	Main predisposing factors
Pseudomembranous	White plaques that easily detach, leaving an erythematous surface	Immunosuppression (HIV, chemotherapy), use of antibiotics or systemic corticosteroids
Acute erythematous	Redness and burning sensation, especially on the dorsum of the tongue	Recent use of antibiotics, use of inhaled corticosteroids
Chronic erythematous (denture stomatitis)	Inflammation under the complete or partial denture, usually in the palate	Continuous use of prostheses, poor hygiene, xerostomia
Chonic hyperplastic	Thick, adherent white plaques that do not detach on scraping or multiple chronic nodules	Smoking, immunosuppression
Angular cheilitis	Fissures and erythema at the corners of the mouth	Ill‐fitting dentures, loss of vertical dimension, nutritional deficiency (iron)
Median rhomboid glossitis	Symmetrical rhomboid or elliptical lesion involving the dorsum of the tongue	Smoking and inhaled corticosteroids
Chronic mucocutaneous candidiasis	Manifests with lesions in the oral cavity and other areas of the body	Immunosuppression

### 2.3 Candidiasis and NACRY

Fungal infections have shown a marked increase in incidence since the 1980s, particularly as a consequence of the HIV pandemic and advances in medical procedures and therapies aimed at treating conditions that were previously lethal, such as organ transplantation and cancer treatments (Armstrong‐James et al. 2014). Although *C. albicans* is the most prevalent and virulent species, several factors, including the widespread use of antifungals (Pathadka et al. 2022), invasive medical devices (such as catheters) (Ma et al. 2013), host conditions (microbiome disruption, immunosuppression, and hospitalization) (Raja 2021), global factors (globalization and climate change) (Garcia‐Solache and Casadevall 2010), and even ecological factors (environmental adaptation and interspecific hybridization) (Gabaldón 2024) have contributed to the emergence of other species known as NACRY, including the current and former *Candida* spp. whose taxonomy was recently changed.

**Figure 1:**
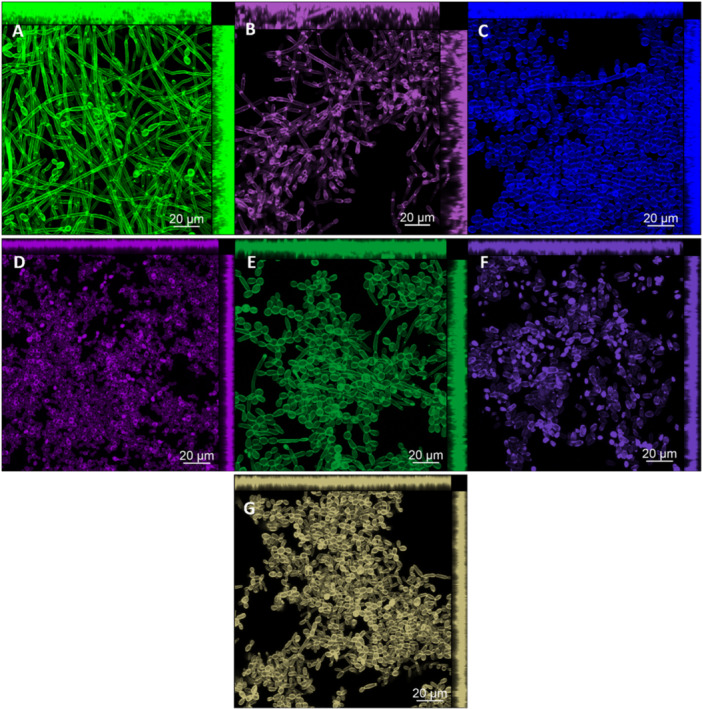
Confocal microscopy images of monospecies biofilms formed by seven *Candida* species for 48 h, stained with Calcofluor White and digitally colored according to their respective colony colors on CHROMagar *Candida*. Above and beside each image is shown the biofilm thickness. Each species is identified: (A) *Candida albicans*, 16 μm thickness, (B) *Pichia kudriavzevii*, 20.69 μm, (C) *Candida tropicalis*, 11.40 μm, (D) *Nakaseomyces glabratus*, 10.82 μm, (E) *Candida dubliniensis*, 14.16 μm, (F) *Meyerozyma guilliermondii*, 11.69 μm, and (G) *Candida parapsilosis*, 10.20 μm. Distinct morphological features can be observed for each species.

**Figure 2:**
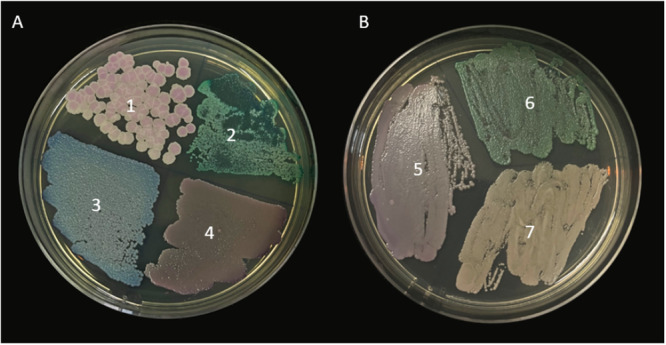
Macroscopic growth of colonies of seven *Candida* species on chromogenic medium (CHROMagar *Candida*), demonstrating phenotypic differences used for presumptive identification. (A) Plate containing four species: (1) *Nakaseomyces glabratus*, (2) *Candida albicans*, (3) *Candida tropicalis*, and (4) *Pichia kudriavzevii*. (B) Plate containing three species: (5) *Meyerozyma guilliermondii*, (6) *Candida dubliniensis*, and (7) *Candida parapsilosis*.

The emergence of NACRY species is strongly associated with host immunosuppression and the indiscriminate use of antifungal agents, which exert selective pressure and favor the emergence of less susceptible species. In this context, *C. dubliniensis* emerged during the HIV pandemic as a species closely related to *C. albicans*, having been isolated from the oral cavity of HIV‐infected patients in Ireland in 1995 (Sullivan et al. 1995). Among the clinically relevant NACRY species in humans are *N. glabratus*, *C. tropicalis*, *C. parapsilosis*, *P. kudriavzevii*, *Clavispora lusitaniae*, *Meyerozyma guilliermondii*, *C. dubliniensis*, and *C. auris*. Figure 1 presents images obtained by confocal laser scanning microscopy of seven *Candida* spp. species cultivated as monospecies biofilms. Figure 2 illustrates the colony growth pattern of these same species on CHROMagar *Candida* chromogenic medium, highlighting differential phenotypic characteristics. NACRY species have increasingly been reported as etiological agents of candidemia in different regions of the world, with geographical variation in prevalence: *N. glabratus* predominates in the United States and in northern and central Europe; *C. tropicalis* is frequently isolated in South America and Asia; and *C. parapsilosis* is commonly reported in South America, Asia, and southern Europe (Falagas et al. 2010). With the exception of *C. auris* (Vila, Sultan, et al. 2020), the other species are also implicated in infections of the oral cavity (Muadcheingka and Tantivitayakul 2015). In general, infections caused by NACRY species represent an important therapeutic challenge due to their reduced susceptibility to antifungal agents (Kołaczkowska and Kołaczkowski 2016).

The pathogenicity of *Candida* spp. is largely determined by its virulence factors, defined as mechanisms and/or molecules produced by the microbial cell that contribute to colonization, tissue invasion, immune evasion, and subsequent host damage. The virulence factors of *C. albicans* are well studied, while those of NACRY are less well documented and typically investigated based on what is known for *C. albicans*. Among these factors, polymorphism is particularly noteworthy, characterized by the ability of the species to switch its morphology from yeast to filamentous forms, such as hyphae and pseudohyphae. This morphological plasticity is closely associated with increased pathogenicity, particularly by facilitating tissue invasion and dissemination within the host (Mba and Nweze 2020). Polymorphism is particularly prominent in *C. albicans* and is considered one of the main determinants of its virulence (Thompson et al. 2011; Lass‐Flörl et al. 2024). Similarly, *C. tropicalis* (Zuza‐Alves et al. 2017) and *C. dubliniensis* (Sullivan et al. 1995) also exhibit polymorphic capacity. The latter is distinguished by forming chlamydospores and by exhibiting lower virulence when compared with other species of the genus. Nevertheless, it is capable of causing superficial infections and, less frequently, invasive disease, in addition to presenting potential for the development of resistance to antifungal agents (Sullivan et al. 2004; Palige et al. 2013).

Other NACRY species exhibit limited or absent morphological plasticity, which may influence their pathogenic behavior. While *C. parapsilosis* and *M. guilliermondii* display predominantly yeast‐like growth with occasional pseudohyphae formation (Kadosh and Mundodi 2020), *N. glabratus* does not produce filaments and grows exclusively as yeast (Rodrigues et al. 2017). *P. kudriavzevii*, in turn, exhibits predominantly yeast‐like morphology, characterized by elongated cells with a cylindrical shape, in contrast to the spherical–oval morphology observed in other species of the genus. Although capable of forming hyphae and pseudohyphae, this species tends to exhibit lower virulence in murine models of systemic infection. This characteristic is evidenced by its inability to induce mortality in immunocompetent mice and by the lower fungal recovery from infected organs when compared with *C. albicans* (Gómez‐Gaviria and Mora‐Montes 2020). Although *C. dubliniensis* can cause superficial and occasionally invasive infections and may develop resistance to antifungals (Sullivan et al. 2004; Palige et al. 2013), its lower pathogenicity has been attributed to evolutionary differences (Moran et al. 2012; Satala, Juszczak, et al. 2022; Gómez‐Gaviria et al. 2024), reduced filamentation capacity (Stokes et al. 2007), and greater susceptibility to polymorphonuclear leukocytes (Vilela et al. 2002). Among emerging fungi, *C. auris* exhibits even more pronounced morphological limitations. Evidence indicates that this species does not produce germ tubes, pseudohyphae, or chlamydospores (Lee et al. 2011; Chowdhary et al. 2014; Borman et al. 2016). However, under specific osmotic stress conditions, such as in media with high salt concentration (Wang et al. 2018), it may exhibit pseudohyphae‐like morphologies, a phenomenon also occasionally observed in biofilms (Sherry et al. 2017). In parallel, comparative genomic analyses support this limitation, demonstrating the absence of the *ECE1* and *HWP1* genes, which are highly expressed in *C. albicans* and strongly associated with hyphae formation (Muñoz et al. 2018).

The ability to adhere to biotic and abiotic surfaces constitutes another key determinant of virulence, representing the initial step in biofilm formation. This process depends on the expression of adhesins, surface proteins capable of recognizing and interacting with molecules present in epithelial and endothelial host cells (Nikou et al. 2019; Lass‐Flörl et al. 2024). Adhesins may confer competitive advantages in specific ecological niches depending on microenvironmental conditions (Zuza‐Alves et al. 2017). In *C. albicans*, the predominant adhesins include the agglutinin‐like sequence (Als) family (Als1–7 and Als9), the hyphal wall protein (Hwp), and Iff/Hyr proteins, whose gene expression is regulated during filamentation (Wijaya et al. 2023). During biofilm formation, different adhesins perform specific functions: Als*1–3* act predominantly in adhesion to abiotic surfaces, whereas Als*2* and *Als3*, together with Hwp*1* and Iff/Hyr proteins, participate in adhesion to biotic substrates (de Souza et al. 2023). *C. dubliniensis*, in turn, shares most members of the *ALS* family, except *ALS3*, an adhesin implicated in host–cell interaction and induction of filamentation (Gómez‐Gaviria et al. 2024). In *C. auris*, the presence of *ALS4* has been described, and its expression has been associated with an increased capacity for adhesion and biofilm formation in this species (Bing et al. 2023). In contrast, *ALS* genes have not been identified in the genome of *P. kudriavzevii* (Gómez‐Gaviria and Mora‐Montes 2020).

With respect to *N. glabratus*, whose morphology is restricted to the yeast form, adhesion and biofilm formation are central determinants of its pathogenicity (Timmermans et al. 2018). Since the initial sequencing of its genome (Dujon et al. 2004), multiple cell wall proteins (Cwps) absent in *Saccharomyces cerevisiae*, a species phylogenetically close to *N. glabratus*, have been described. Subsequently, genome sequencing of species within the genus *Nakaseomyces* (Gabaldón et al. 2013) indicated greater phylogenetic proximity between former *C. glabrata* and these species than with *S. cerevisiae*, as well as the sharing of several genes of the Epa (epithelial adhesin) family (Gabaldón et al. 2013). The adhesins of *N. glabratus* are classified into seven groups (I–VII) (López‐Fuentes et al. 2018). Group I (Epa) represents the largest and most studied family, with 18 and 23 genes described in the strains CBS138 and BG2, respectively (Dujon et al. 2004; Kaur et al. 2005). Group II comprises the Pwp family (cell wall proteins containing a PA14 domain), consisting of at least seven members (Pwp1–7), which, like Epa, contain the conserved anthrax protective antigen (PA14) domain at their N‐terminus. The remaining groups (III–VII) include the Awp family (adhesin‐like wall proteins), with 13 members (Awp1–13), as well as other Cwps with limited similarity at the N‐terminal region (de Groot et al. 2013; López‐Fuentes et al. 2018; Timmermans et al. 2018).

Regarding *C. tropicalis*, a high capacity for adhesion to both biotic and abiotic surfaces has been observed, often exceeding that of *C. albicans*, a characteristic correlated with the presence of 16 genes of the *ALS* family and the *HWP1* gene, also present in *C. albicans* (Dos Santos and Ishida 2023; de Souza et al. 2023). In addition, Iff/Hyr proteins expressed during the hyphal phase have been described, capable of interacting with components of the host extracellular matrix and showing high similarity to homologs in *C. albicans* (Kozik et al. 2015). In *C. parapsilosis*, the *HWP1* gene plays a crucial role in initial adhesion during biofilm formation (Wan Harun et al. 2013). This species shows affinity for oral epithelial cells comparable to that observed in *C. albicans* (Panagoda et al. 2001), in addition to expressing Cwps and proteins analogous to the Als family in its pseudohyphae, which promotes adhesion to both biotic and abiotic surfaces (Kozik et al. 2015; Tóth et al. 2019). *C. parapsilosis* is frequently isolated from the skin and hands (Yapar 2014), facilitating colonization of catheters and medical devices and consequently increasing the risk of candidemia, particularly in neonates receiving parenteral nutrition in ICUs (Bonassoli et al. 2005; Pammi et al. 2013). In *M. guilliermondii*, comprehensive bioinformatic analyses have indicated the presence of the Als family (MgAls), suggesting that these proteins may play a relevant role in cellular adhesion and surface colonization processes (S. J. Lim et al. 2025). Finally, *C. lusitaniae* exhibits greater hydrophobicity than *C. albicans*, but lower efficiency in adhesion to human epithelial cells, a fact associated with the lower virulence of this species (Muadcheingka and Tantivitayakul 2015; Mendoza‐Reyes et al. 2022). Nevertheless, its ability to colonize inert surfaces favors biofilm formation on medical devices (Dorko et al. 1999). Genomic studies have identified orthologs related to thermotolerance (*HSP60* and *HSP104*) and immune evasion (*HGT1* and *MSB2*), which contribute to the adaptation of *C. lusitaniae* to host‐imposed conditions (Mendoza‐Reyes et al. 2022).

In addition to polymorphism and adhesion capacity, the secretion of extracellular hydrolytic enzymes is an important virulence factor in NACRY, encompassing hemolysins, phospholipases, and proteinases that act in an integrated manner during pathogenesis and infection progression. These enzymes not only promote tissue invasion and dissemination but also play a decisive role in the transition from the commensal state to the invasive phenotype, particularly in immunocompromised hosts. Hemolysins are notable for promoting erythrocyte lysis, thereby enabling the acquisition of iron, an element essential for fungal metabolism (Mroczyńska and Brillowska‐Dąbrowska 2021). Complementarily, phospholipases hydrolyze phospholipids in host cell membranes, compromising their integrity and facilitating both tissue invasion and microbial dissemination (Ghannoum 2000). Secreted proteinases, in turn, degrade structural proteins, such as collagen, keratin, and mucin, in addition to acting on immunoglobulins, thereby contributing to immune evasion (Naglik et al. 2003; Mroczyńska and Brillowska‐Dąbrowska 2021). Additionally, lipases hydrolyze lipids, providing sources of carbon and energy for the pathogenic microorganism (Hube et al. 2000).

Regarding NACRY species, several studies have reported that hydrolytic enzymes are important virulence factors. Species such as *N. glabratus* (Staniszewska 2020; Gaffar et al. 2025), *P. kudriavzevii* (Musinguzi et al. 2024), and *C. auris* (Satala, Juszczak, et al. 2022; Gaffar et al. 2025) exhibit proteinase and phospholipase activities. In *C. dubliniensis*, the reported enzymatic activity is mainly associated with the production of proteinases (Vidotto et al. 2004). In contrast, *C. tropicalis* (Staniszewska 2020) and *C. parapsilosis* (Staniszewska 2020; Gaffar et al. 2025) display a broader enzymatic repertoire that includes, in addition to proteinases and phospholipases, the production of lipases. It is also noteworthy that, beyond the secretion of extracellular hydrolytic enzymes (Schaller et al. 2005), *C. albicans* produces candidalysin, a toxic peptide associated with the hyphal form that acts through pore formation in the plasma membrane, promoting calcium influx and cell lysis, while simultaneously activating “danger response” signaling pathways and modulating epithelial immunity (Moyes et al. 2016). Candidalysin has also been implicated in neutrophil recruitment and increased virulence in systemic infection models and is considered a critical factor in endothelial damage and in the induction of pro‐inflammatory cytokines responsible for phagocyte activation (Swidergall et al. 2019). On the other hand, *C. tropicalis* and *C. dubliniensis* harbor orthologs of the ECE1 gene, which is responsible for candidalysin production. It has been demonstrated that these candidalysin variants exhibit greater cytolytic and immunomodulatory activity in oral epithelial cells in vitro than the candidalysin produced by *C. albicans* (Richardson et al. 2022). It has also been shown that candidalysin variants with different amino acid sequences are present in different strains of *C. albicans*, *C. tropicalis*, and *C. dubliniensis*, and that these variants exhibited distinct abilities to cause damage and elicit immune responses in epithelial cells (Wickramasinghe et al. 2024).

Finally, among the virulence factors discussed, extracellular matrix (ECM) synthesis represents a key factor in infection persistence and therapeutic refractoriness associated with biofilms. The importance of this component was initially demonstrated by pioneering studies that reported a significant reduction in the efficacy of amphotericin B under culture conditions that favored ECM synthesis (Al‐Fattani and Douglas 2004, 2006; Vediyappan et al. 2010). Subsequent genetic and pharmacological analyses confirmed that the mannan–glucan complex acts in a conserved manner across species, promoting the sequestration of multiple classes of antifungals and conferring protection to the associated microbial community (Mitchell et al. 2013). Corroborating these findings, other authors demonstrated that mature biofilms of *C. albicans* retain nearly all radiolabeled triazoles and fluconazole within the ECM (Nett et al. 2010).

In addition to mediating antifungal tolerance, the ECM contributes to the structural maturity and robustness of the biofilm, with a composition that varies among species and, consequently, influences their pathogenic potential (Wijaya et al. 2023). In *C. albicans*, ECM is composed of carbohydrates, proteins, lipids, and extracellular DNA (Zarnowski et al. 2014), as well as hexosamine, phosphorus, and uronic acid (Al‐Fattani and Douglas 2006). The carbohydrate fraction is predominantly composed of glucose and the mannan–glucan complex, formed by α‐1,6‐mannan with α‐1,2 branches linked to unbranched α‐1,6‐glucan, a structure directly associated with fluconazole sequestration and antifungal resistance (Zarnowski et al. 2014; Massey et al. 2023). Lipids include neutral and polar glycerolipids as well as sphingolipids (Zarnowski et al. 2014).

In *C. tropicalis*, a higher proportion of hexosamine and uronic acid is observed, accompanied by lower concentrations of carbohydrates, proteins, and phosphorus compared with *C. albicans* (Al‐Fattani and Douglas 2006). Nevertheless, its biofilms exhibit greater resistance to detachment (Zarnowski et al. 2014; Wijaya et al. 2023). In this species, cellular adhesion is regulated by genes of the *ALS* family (*ALS1–16*), whereas biofilm maturation depends on the transcription factor EFG1, which is also involved in filamentation regulation and performs functions similar to those described in *C. albicans* (Araújo et al. 2017). Although considered less virulent than *C. albicans*, *P. kudriavzevii* has clinical relevance due to its intrinsic resistance to fluconazole and reduced susceptibility to other antifungal classes, including polyenes such as amphotericin B and echinocandins (Yadav et al. 2012; Nguyen et al. 2024). Similarly, *M. guilliermondii* exhibits low clinical virulence, associated with limited biofilm formation, reduced susceptibility to azoles and echinocandins (Marcos‐Zambrano et al. 2017), and weak induction of pro‐inflammatory cytokines, with its cell wall mannans playing a determining role in immune recognition by monocytes (Navarro‐Arias et al. 2016).

In addition to the virulence factors previously described, immune evasion mechanisms and the ability to survive and proliferate at physiological temperatures contribute substantially to pathogenicity. In this context, *C. auris* emerges among NACRY species. Evidence indicates that *C. auris* is capable of inhibiting neutrophil activity (Johnson et al. 2018) and inducing increased expression of PD‐1 in T lymphocytes, as well as its ligand PD‐L1 in macrophages from infected mice, thereby favoring infection persistence (Wurster et al. 2022). Regarding thermotolerance, *C. auris* demonstrates optimal growth at 37°C and maintains viability up to 42°C (Satoh et al. 2009; Borman et al. 2016). Additionally, *C. auris* isolates have been observed to secrete greater amounts of aspartyl proteinases (Saps) at 42°C than *C. albicans* under the same conditions, suggesting not only a high degree of adaptation to thermal stress but also the preservation of virulence attributes at elevated temperatures (Wang et al. 2018). Alongside thermal tolerance, *C. auris* also exhibits high osmotolerance, a characteristic associated with the formation of compact cellular aggregates with low dispersion, a phenotype that may favor the persistence of the microorganism on both biotic and abiotic surfaces, including hospital environments, and potentially increase its transmission and resistance to infection control measures (Osei Sekyere 2018; Kean et al. 2018).

The combination of immune evasion mechanisms and a remarkable capacity to survive under adverse environmental conditions renders *C. auris* a pathogen of increasing clinical relevance. This scenario highlights the need for in‐depth investigations into the role of NACRY species in the etiology of candidemia, with particular emphasis on hospital environments, where the persistence and dissemination of these microorganisms represent a significant public health challenge.

In general, *Candida* species exhibit distinct repertoires of virulence factors, in which the presence, absence, or modulation of specific mechanisms contribute to distinct pathogenic strategies. Whereas *C. albicans* combines multiple classical virulence determinants, such as polymorphism, candidalysin production, secretion of hydrolytic enzymes, and robust biofilm formation, several NACRY exhibit partially distinct repertoires, often characterized by a lower capacity for filamentation, but compensated by alternative mechanisms of adhesion, biofilm formation, or immune evasion. A comparative overview of these main virulence factors among the species discussed is presented in Table 2.

**Table 2:** Comparison of virulence factors among *Candida albicans*, non‐*albicans Candida*, and related yeasts (NACRY).

Species	Morphology	Adhesion	Enzymes/toxins	Extracellular matrix (biofilm)
*C. albicans*	Polymorphism (yeast, hyphae, and pseudohyphae)^1^ ^,^ ^2^	Als family (Als1‐7 and Als9), HWP1, and Iff/Hyr^16^	Hemolysins, phospholipases, proteinases, lipases, and candidalysin^30^ ^,^ ^31^ ^,^ ^32^	Glycoproteins, carbohydrates, lipids, nucleic acids, hexosamine, phosphorus, and uronic acid^38^ ^,^ ^39^
*Candida dubliniensis*	Polymorphism (yeast, hyphae, and pseudohyphae) with chlamydospore formation^3^ ^,^ ^4^ ^,^ ^5^	ALS family (the absence of ALS3)^17^	Candidalysin variants and proteinases^33^ ^,^ ^34^ ^,^ ^35^	—
*Candida tropicalis*	Polymorphism (yeast, hyphae, and pseudohyphae)^6^	16 ALS family genes, HWP1, and Iff/Hyr^18^ ^,^ ^19^ ^,^ ^20^	Candidalysin variants, phospholipases, proteinases, and lipases^32^ ^,^ ^34^ ^,^ ^35^	Hexosamine and uronic acid, lower concentrations of carbohydrates, proteins, and phosphorus compared with *C. albicans* 39
*Nakaseomyces glabratus*	Exclusively yeast^6^	Epa, Pwp, and Awp families^21^ ^,^ ^22^ ^,^ ^23^ ^,^ ^24^ ^,^ ^25^	Phospholipases and proteinases^30^ ^,^ ^32^	Proteins and carbohydrates, and little is known about the rest in the literature^40^
*Candida parapsilosis*	Predominantly yeast with occasional pseudohyphae formation^7^	HWP1, Cwps, and Als‐like proteins^26^ ^,^ ^21^ ^,^ ^27^	Phospholipase, proteinases, and lipases^30^ ^,^ ^32^	Carbohydrates and lower concentrations of proteins compared with other species^40^
*Pichia kudriavzevii*	Predominantly yeast with elongated cells and occasional hyphae/pseudohyphae^8^	No ALS genes identified^8^	Phospholipases and proteinases^36^	Robust biofilm formation^41^
*Clavispora lusitaniae*	Predominantly yeast^9^	—	—	—
*Meyerozyma guilliermondii*	Predominantly yeast^7^	Als family (MgAls)^28^	—	Limited biofilm formation^42^
*Candidozyma auris*	Predominantly yeast with occasional pseudohyphae formation^10^ ^,^ ^11^ ^,^ ^12^ ^,^ ^13^ ^,^ ^14^ ^,^ ^15^	ALS4 gene^29^	Phospholipases and proteinases^30^ ^,^ ^37^	Mannan–glucan polysaccharides^16^

*Note:* The table summarizes the main virulence‐associated traits reported for these species, including morphological characteristics, major adhesins, secreted enzymes or toxins, and components of the biofilm matrix described in the literature. When information was not available, the field is indicated with a dash (–). References supporting each characteristic are listed below.

^1^ Thompson et al. (2011).

^2^ Lass‐Flörl et al. (2024).

^3^ Sullivan et al. (1995).

^4^ Sullivan et al. (2004).

^5^ Palige et al. (2013).

^6^ Zuza‐Alves et al. (2017).

^7^ Kadosh and Mundodi (2020).

^8^ Gómez‐Gaviria and Mora‐Montes (2020).

^9^ Mendoza‐Reyes et al. (2022).

^10^ Lee et al. (2011).

^11^ Chowdhary et al. (2014).

^12^ Borman et al. (2016).

^13^ Wang et al. (2018).

^14^ Sherry et al. (2017).

^15^ Muñoz et al. (2018).

^16^ Wijaya et al. (2023).

^17^ Gómez‐Gaviria et al. (2024).

^18^ Dos Santos and Ishida (2023).

^19^ de Souza et al. (2023).

^20^ Kozik et al. (2015).

^21^ Gabaldón et al. (2013).

^22^ Dujon et al. (2004).

^23^ Kaur et al. (2005).

^24^ López‐Fuentes et al. (2018).

^25^ Timmermans et al. (2018).

^26^ Wan Harun et al. (2013).

^27^ Tóth et al. (2019).

^28^ S. J. Lim et al. (2025).

^29^ Bing et al. (2023).

^30^ Gaffar et al. (2025).

^31^ Schaller et al. (2005).

^32^ Staniszewska (2020).

^33^ Vidotto et al. (2004).

^34^ Richardson et al. (2022).

^35^ Wickramasinghe et al. (2024).

^36^ Musinguzi et al. (2024).

^37^ Satala, Juszczak, et al. (2022).

^38^ Zarnowski et al. (2014).

^39^ Al‐Fattani and Douglas (2006).

^40^ Ciurea et al. (2020).

^41^ Jain et al. (2025).

^42^ Marcos‐Zambrano et al. (2017).

### 2.4 Oral Microbiome and Candidiasis

The oral microbiome encompasses a highly complex microbial community (the greatest community after the gut) and plays a dynamic ecosystem in constant interaction with the host (Deo and Deshmukh 2019). Different environmental conditions in the mouth create unique habitats, such as teeth, tongue, gingival sulcus, cheek, floor, hard and soft palate coated by saliva, where specific microbial communities adhere and thrive as biofilms (Di Stefano et al. 2022). Oral microbiome involves bacteria, fungi, viruses, archaea, and protozoa, but currently only oral bacteriome is well described with more than 700 prokaryotic species (Dewhirst et al. 2010; Deo and Deshmukh 2019), while oral mycobiome is less explored despite its relevance in health and disease (Defta et al. 2024). Very few investigations have described the archaeome found in periodontal sites (Belmok et al. 2020) and dental caries (Dame‐Teixeira et al. 2025). In a state of homeostasis, the oral microbiome maintains ecological balance and a symbiotic relationship with both the host and its local environment. However, local and systemic conditions, such as immunosuppression, alter this symbiotic interaction, enabling opportunistic pathogens to prosper and resulting in clinical implications for oral health and, sometimes, systemic health too (Di Stefano et al. 2022). The presence of the yeast *C. albicans*, combined with host susceptibility, is a key factor in the development of oral candidiasis. However, evidence suggests that this condition is not solely a fungal infection but rather a polymicrobial infection involving specific interactions between *C. albicans* and certain bacterial species, which may modulate its transition from a commensal to a pathogenic state (Xu, Jenkinson, et al. 2014).

In vitro models have demonstrated that *C. albicans* is capable of forming synergistic biofilms with bacterial species, such as *Streptococcus oralis*, *Streptococcus sanguinis*, and *Streptococcus gordonii*, enhancing its invasive capacity in oral and esophageal mucosal models (Diaz et al. 2012). These findings suggest that microbial interactions play a significant role in the fungal pathogenicity process. Additionally, the presence of *S. oralis* has been shown to not only intensify biofilm formation and *C. albicans* dissemination but also promote the expression of pro‐inflammatory cytokines, contributing to an immune environment that favors infection (Xu, Sobue, et al. 2014). On the other hand, certain bacterial species, such as the periodontal pathogen *Aggregatibacter actinomycetemcomitans*, are capable of secreting molecules that inhibit both biofilm formation and the filamentation process of *C. albicans*, suggesting a potential negative modulatory effect of these bacteria on fungal pathogenicity (Bachtiar et al. 2014).

Within this complex scenario of interkingdom interactions, *C. albicans* plays a critical role in the formation of mixed pathogenic biofilms, as it provides mechanical support for the adhesion and colonization of both Gram‐positive and Gram‐negative bacteria, thereby creating a favorable substrate for microbial establishment (Krom et al. 2014; Janus et al. 2016; Bhardwaj et al. 2020). Specifically, the fungal cell wall is composed of a complex network of structural polysaccharides, adhesion proteins, and surface receptors that play a key role in mediating interactions with various species in the oral microbiome (Kashyap et al. 2024). Evidence indicates that *S. gordonii*, a commensal bacterium of the oral cavity that plays a fundamental role in dental plaque formation, interacts directly with *C. albicans* via proteins located in the fungal cell wall, promoting hyphal development (Bamford et al. 2009). Furthermore, studies have shown that the interaction among *C. albicans*, *Porphyromonas gingivalis*, *S. aureus*, and specific *Streptococcus* species, such as *S. sanguinis* and *S. oralis*, results in the formation of highly structured biofilms, suggesting a significant degree of functional coordination among these microorganisms in the oral environment (Kim and Koo 2020).

Despite the influence of multiple factors, microbial biofilms dominated by *C. albicans* play a decisive role in the development of denture stomatitis, due to both its strong adherence to acrylic resin and its ability to colonize oral mucosal surfaces (Gendreau and Loewy 2011). These features, combined with the expression of various virulence factors (Yoo et al. 2020; Satala, Gonzalez‐Gonzalez, et al. 2022), also account for the frequent association of *C. albicans* with other oral infections reported in the literature. Evidence indicates that *C. albicans* and *Streptococcus mutans* interact synergistically in the formation of thick, acidic, and highly cariogenic biofilms (Koo and Bowen 2014), which may contribute to more extensive and rapidly progressing carious lesions (Koo et al. 2018). A systematic review and meta‐analysis revealed that *C. albicans* is the most commonly isolated fungal species from infected root canals (Mergoni et al. 2018), particularly in persistent or refractory endodontic infections that do not respond to root canal treatment (Yoo et al. 2020). In an in vitro model, *P. gingivalis*, a red‐complex bacterium widely recognized for its high virulence in periodontal disease, was found to promote the adhesion and hyphal formation of *C. albicans* on artificial surfaces. In turn, the biofilm formed by this yeast supported colonization by the bacterial pathogen, indicating a mutual interaction that favored the development of both microorganisms (Bartnicka et al. 2019). In the context of peri‐implantitis, *C. albicans* may have the ability to adhere to implant surfaces and form biofilms harboring a complex microbial community, thereby contributing to the persistence and severity of infection (Souza et al. 2022; Di Spirito et al. 2024). Additionally, *C. albicans* has been associated with oral carcinogenesis, being identified as a microorganism capable of activating oncogenes, enhancing premalignant features, and participating in various stages of oral cancer development (Vadovics et al. 2021).

The mechanisms that confer competitive advantages during microbial colonization include not only metabolic cooperation, modulation of signaling pathways, and horizontal gene transfer but also nutrient competition (Marsh and Zaura 2017). In this context, sucrose acts as an environmental factor that promotes coaggregation between *C. albicans* and *Streptococcus* species, inducing the expression of virulence‐related genes in both microorganisms (Ellepola et al. 2019). In biofilms composed of *C. albicans* and *S. mutans*, bacterial microcolonies have been observed surrounding fungal cells, all embedded within a polysaccharide‐rich ECM (Kim and Koo 2020). Additional findings indicate that, in the presence of sucrose, there is a significant increase in adhesion between *S. mutans* and *C. albicans*, while in the absence of this carbohydrate, a stronger binding affinity was noted between *C. albicans* and *S. gordonii* (Wan et al. 2021). In immunocompetent murine models, sucrose supplementation increased bacterial load on the lingual surface, reduced oral bacteriome alpha diversity, and increased the abundance of native oral *Streptococcus* species (Bertolini et al. 2021).

In addition to its association with oral bacterial species, *C. albicans* also interacts with medically relevant bacterial species responsible for systemic infections, some of which may be transient inhabitants of the oral cavity. Many of these bacteria are currently listed among the global priority pathogens reported by the World Health Organization (WHO), including methicillin‐resistant *S. aureus* (Howden et al. 2023) and carbapenem‐resistant *Pseudomonas aeruginosa* (Reyes et al. 2023). The interaction between *S. aureus* and *C. albicans* has been extensively documented, revealing a synergism that enhances virulence and complicates treatment. *S. aureus* is a pathogenic Gram‐positive bacterium responsible for severe hospital‐ and community‐acquired infections, especially when caused by its resistant form (methicillin‐resistant *S. aureus*) (Howden et al. 2023). *C. albicans* provides a tridimensional structure through its hyphae, facilitating the adhesion and persistence of *S. aureus* during biofilm formation (Peters et al. 2010) via interaction with the fungal adhesin Als3p present in its cell wall (Schlecht et al. 2015). In this context, evidence shows that the polymicrobial biofilm, structured and protected by a dense ECM produced by *C. albicans* and rich in β‐1,3‐glucan (Kong et al. 2016), contributes to the protection of *S. aureus* against vancomycin, whereas the growth and susceptibility of *C. albicans* to amphotericin B remain unaffected (Harriott and Noverr 2009). Moreover, *C. albicans* has been shown to induce and promote the growth of *S. aureus* small‐colony variants with increased expression of virulence factors (Arévalo‐Jaimes and Torrents 2024), which are linked to activation of quorum‐sensing systems (a density‐dependent, microbial cell‐to‐cell communication process that uses signaling molecules to coordinate gene expression collectively), increased α‐toxin production, and, consequently, tissue destruction and modulation of the host immune response (Todd et al. 2019).

The interaction between *P. aeruginosa* and *C. albicans* is also well documented in nosocomial infections associated with medical devices (Pierce 2005) and has been observed in urinary and gastrointestinal tract infections, chronic wounds (Fourie and Pohl 2019), and within the pulmonary environment, particularly in patients with cystic fibrosis (Fourie et al. 2016) and ventilator‐associated pneumonia (Azoulay et al. 2006). Both *P. aeruginosa* and *C. albicans* can form biofilms on medical device surfaces, as demonstrated in in vitro models. From this perspective, evidence shows that dual‐species biofilms formed by these microorganisms accumulate greater biomass than single‐species cultures when grown under flow conditions (Kasetty et al. 2021), suggesting a higher propensity for adhesion to urinary catheters (Joshi et al. 2025). Conversely, phenazines produced by *P. aeruginosa* can inhibit biofilm formation, filamentation, and affect colony morphology of *C. albicans* by disturbing respiratory metabolism (Morales et al. 2013), while the fungus can enhance bacterial biofilm development in vitro, possibly due to increased production of alginate, an ECM component of *P. aeruginosa*, and exacerbate bacteremia in mice (Phuengmaung et al. 2020). Similarly, additional studies have demonstrated an antagonistic relationship between these species, in which *P. aeruginosa* is capable of eliminating *C. albicans* hyphae but not yeast cells (Brand et al. 2008), as well as increasing the fungal susceptibility to amphotericin B as duas‐species biofilm, leading to fungal cell death through oxidative stress induced by phenazines produced by *P. aeruginosa* (Alam et al. 2023). Therefore, the synergism or antagonism observed between *C. albicans* and *P. aeruginosa* seems to be related to the growth conditions during biofilm formation. Table 3 summarizes the relationship between *C. albicans* and oral bacteria, as well as medically relevant bacteria.

**Table 3:** Relationships between bacterial species and *Candida albicans* described in the literature. Microbial interactions may range from synergistic to antagonistic, depending on the phenotypic and genetic characteristics of the microorganisms involved, as well as on environmental conditions. References correspond to studies that characterized these interactions.

Species	Classification	Type of interaction	Environment/clinical context	Mechanism involved	References
*Streptococcus orais*	Gram‐positive	Synergism	Oral biofilm	Formation of synergistic biofilms and expression of pro‐inflammatory cytokines	Xu, Sobue, et al. (2014)
					Kim and Koo (2020)
*Streptococcus sanguinis*	Gram‐positive	Synergism	Oral biofilm	Formation of synergistic biofilms	Diaz et al. (2012)
					Kim and Koo (2020)
*Streptococcus gordonii*	Gram‐positive	Synergism	Oral biofilm and *C. albicans* filamentation	Formation of synergistic biofilms and stimulation of *C. albicans* filamentation	Diaz et al. (2012)
					Bamford et al. (2009)
*Aggregatibacter actinomycetemcomitans*	Gram‐negative	Antagonism	Oral biofilm and *C. albicans* filamentation	Secretion of inhibitory molecules against biofilm formation and *C. albicans* filamentation	Bachtiar et al. (2014)
*Porphyromonas gingivalis*	Gram‐negative	Synergism	Oral biofilm and *C. albicans* filamentation	Formation of synergistic biofilms and stimulation of *C. albicans* filamentation	Kim and Koo (2020)
					Bartnicka et al. (2019)
*Staphylococcus aureus*	Gram‐positive	Synergism	Oral biofilm and hospital/community‐acquired infections	Formation of synergistic biofilms and virulence enhancement through quorum‐sensing activation	Kim and Koo (2020)
					Arévalo‐Jaimes and Torrents (2024)
					Todd et al. (2019)
*Streptococcus mutans*	Gram‐positive	Synergism	Oral biofilm	Formation of thick, acidic, and highly cariogenic oral biofilms	Koo and Bowen (2014)
*Pseudomonas aeruginosa*	Gram‐negative	Synergism and antagonism	Nosocomial infections related to medical devices, urinary and gastrointestinal tract infections, chronic wounds, cystic fibrosis, and ventilator‐associated pneumonia	Formation of thick biofilms on medical device surfaces (synergism); *P. aeruginosa* can inhibit *C. albicans* biofilm formation, while *C. albicans* can enhance bacterial biofilm formation (antagonism)	Pierce (2005)
					Fourie and Pohl (2019)
					Fourie et al. (2016)
					Azoulay et al. (2006)
					Morales et al. (2013)
					Phuengmaung et al. (2020)

In patients using removable dentures, the oral cavity constitutes a favorable environment for *C. albicans* colonization, especially under poor oral hygiene conditions and wearing dentures continuously during sleep (Iinuma et al. 2015; T. W. Lim et al. 2024). Such conditions promote the formation of structurally organized fungal biofilms with high potential for persistence and pathogenicity (Malinovská et al. 2023). The presence of dentures in the oral cavity reduces the oxygen and saliva on the palate, creating an anaerobic microenvironment that favors the growth of anaerobic bacteria (Sakima et al. 2022). In this context, chronic aspiration of these microorganisms from denture biofilm by individuals with dysphagia, gastroesophageal reflux, or advanced age may facilitate the penetration of bacteria and *C. albicans* into the lower respiratory tract (Xu et al. 2024). It was demonstrated that denture wearing overnight doubles the risk of pneumonia in elderly people aged 85 or over (Iinuma et al. 2015). Although aspiration pneumonia has classically been attributed to bacterial pathogens (DiBardino and Wunderink 2015; Twigg et al. 2023), evidence suggests that, in certain clinical scenarios, *C. albicans* may also play a relevant role in this infectious process (Meena and Kumar 2022; Xu et al. 2024), particularly in immunocompromised patients. Investigations indicate that the fungus may act opportunistically and synergistically in the pathogenesis of aspiration pneumonia, being detected at high concentrations in respiratory samples, often in association with pathogenic bacteria, and identified in up to 16.1% of cases (Moss and Musher 2021; Xu et al. 2024). Once in the lungs, *C. albicans* can penetrate the alveolar epithelium through the secretion of candidalysin, a cytolytic fungal peptide toxin that plays a significant role in both epithelial membrane damage (Moyes et al. 2016) and the inflammatory response observed in fungal biofilms (Naglik et al. 2019), triggering activation of the MAPK and NF‐kB pathways and leading to the production of pro‐inflammatory cytokines, such as interleukin‐8 (IL‐8) and IL‐1β (Wronowska et al. 2025). Additionally, studies have reported the presence of *C. albicans* in necrotizing lung lesions associated with *Candida* esophagitis and gastritis, reinforcing the hypothesis that the pulmonary infection originates from aspiration in patients without apparent immunosuppression (Jackson et al. 2023). A systematic review showed that adequate oral healthcare, such as tooth brushing after meals, denture hygiene once a day, and professional oral care once a week, reduced the risk of aspiration pneumonia, including the risk of death, in frail elderly patients (van der Maarel‐Wierink et al. 2013). These findings highlight the importance of oral healthcare strategies in patients at increased risk of aspiration pneumonia (Tada and Miura 2012; Müller 2015), since the presence of *C. albicans* in the oral cavity and in denture biofilms may represent a potentially relevant factor in the pathology of aspiration pneumonia.

### 2.5 Animal Model of Oral Candidiasis

Animal models are important in medical research, allowing investigation of disease pathogenesis and pathogen–host interactions, as well as the evaluation of aspects that cannot be ethically or practically studied in humans, such as histopathological changes in infected tissues. Nowadays, the animal model used for oral candidiasis in rodents is very well established. However, rodents were not the first model used to reproduce oral candidiasis as observed in humans, since the first investigations published in the 1970s used monkey as an animal model (Budtz‐Jorgensen 1971; Olsen and Haanaes 1977). However, due to high costs and difficult maintenance, other animal models were employed, such as hamsters (McMillan and Cowell 1985) and rabbits (Walsh et al. 2000), and currently rodents (rats and mice) have been widely used due to their reproducibility, low cost, easy handling, and maintenance (L. P. Samaranayake and Samaranayake 2001).

There is robust evidence that *C. albicans* is able to colonize the oral cavity of rats and mice under immunosuppression with or without local antibiotics, resulting in oral lesions after the yeast is inoculated in the mouth (Li et al. 2025; L. P. Samaranayake and Samaranayake 2001). While mice do not harbor *Candida* in the oral cavity (L. P. Samaranayake and Samaranayake 2001; Phillips and Balish 1966; Lacasse et al. 1990), there is a controversy regarding whether *Candida* spp. is a natural resident of the mouth of rats, because there are review papers reporting that *Candida* spp. do not colonize rats (Li et al. 2025) or may be a transient commensal in the oral cavity of rats (L. P. Samaranayake and Samaranayake 2001). However, there is no evidence demonstrating that rats harbor *Candida* spp. as natural residents of their oral mucosa. Hence, researchers must evaluated the presence of *Candida* spp. in the oral cavity of rats prior to any fungal inoculation and, if *Candida* is detected, such animals should be excluded.

**Figure 3:**
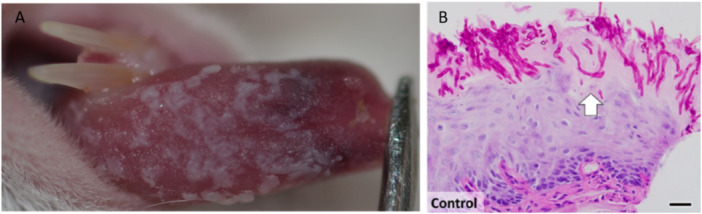
(A) Representation of the experimental model of oral candidiasis in mice, showing white patches on the dorsum of the tongue through the process of immunosuppression and inoculation of *Candida albicans*. (B) Histological section of the tongue of a mouse from the control group (infected animals that did not receive any treatment). Reproduced from Sakima et al. (2018), *Molecules*, DOI: https://doi.org/10.3390/molecules23082075. Creative Commons Attribution License (https://creativecommons.org/licenses/by/4.0/).

The pioneering studies reporting oral lesions in a murine model of oral candidiasis were developed by Kamai et al. (2001) and Takakura et al. (2003), who reported that immunosuppression, performed by injections of prednisolone or cortisone, is a crucial requirement for the development of oral candidiasis. Then, *C. albicans* is inoculated in the murine oral cavity by swabbing a cotton swab immersed in a fungal suspension on the dorsum of the tongue. Some studies also administered tetracycline in the drinking water in order to disrupt the oral bacterial community and favor *Candida* colonization (Takakura et al. 2003; Solis and Filler 2012; Mima et al. 2023). Other investigations add suspensions of *C. albicans* to the drinking water to keep a high fungal load in the mouth (Dongari‐Bagtzoglou et al. 2009). This method results in oral lesions characterized by white patches or pseudomembranes covering from 21% to 90% of the dorsum of the tongue (Figure 3A) after 5–7 days of inoculation, with recovery rates of 10^5−6^ CFU of *C. albicans* (Takakura et al. 2003; Solis and Filler 2012; Mima et al. 2010; Mima et al. 2023). The histopathological analysis of the infected tongue usually shows yeast and hyphae of *C. albicans* on the keratinized layer of the epithelium with or without invasion, disorganization and/or destruction of the epithelial layers, loss of filiform papillae and mild inflammatory response in the connective tissue mediated by monocytes and neutrophils (Figure 3B) (Takakura et al. 2003; Mima et al. 2010; Costa et al. 2012). This model has been widely used for investigating the fungal virulence, pathogenesis, host immune response and bacteria–fungus interaction (Villar and Dongari‐Bagtzoglou 2008; Dongari‐Bagtzoglou et al. 2009; Diaz et al. 2014) as well as therapeutic approaches for this infection (Farah and Ashman 2005; Conti and Gaffen 2015). Recent studies extended the infection period by increasing the frequency of immunosuppression performed in the animals, allowing the evaluation of multiple therapeutic sessions (Carmello et al. 2016; Sakima et al. 2018; Hidalgo et al. 2019).

Other experimental models have been used to reproduce oral candidiasis, such as sialoadenectomy, defined as the surgical removal of the salivary glands. This procedure induces xerostomia and consequently favors the colonization of *C. albicans* of the oral mucosa of rats, thereby allowing for the experimental reproduction of oral candidiasis (Jorge et al. 1993).

This model has also been described in mice; however, in that study, no clinical evaluations of the lesions were reported, only histopathological analyses confirming the presence of hyphal invasion of the epithelium (Totti et al. 2004).

Recently, the sialoadenectomy model was used in rats, and topical inoculation with a swab soaked in a suspension of *C. albicans* was performed for 2 consecutive days. In addition to microbiological confirmation of colonization by culture, the authors inspected macroscopically the tongue daily, observed lesions, and conducted histological analysis using the O'Grady and Reade scale, a semiquantitative scale that assesses the degree of hyphal invasion of the epithelium, which confirmed the presence of hyphae invading the epithelial tissue (Pérez‐Sayáns et al. 2021).

This model is considered effective as an alternative to systemic immunosuppression, allowing the evaluation of colonization and tissue invasion (Jorge et al. 1993; Pérez‐Sayáns et al. 2021). However, it has the disadvantage of increased surgical complexity, especially in mice due to their small size, rendering the technique more delicate and difficult to reproduce (Costa et al. 2013).

The mucositis model is also used to reproduce oral candidiasis in rodents, based on the inoculation of *C. albicans* into animals previously subjected to chemotherapy, usually with agents such as cisplatin and 5‐fluorouracil, to simulate the immunological conditions of oncological patients as an alternative to traditional immunosuppression. Mucositis can be induced in different ways. The most commonly used method involves chemical induction, usually through the application of acetic acid to the oral mucosa (Y. S. Lim et al. 2016; Fujisawa et al. 2003; Katagiri et al. 2018). Another alternative is physical induction, as in the model described by Sonis et al. (1990), which consists of mechanical irritation of the mucosa.

Histopathological analyses reveal disruption of the basal epithelial layer, with fungi invading the subepithelium and intravascular hyphae. In addition, this model reproduces white plaques on the tongue, typical of the pseudomembranous form of oral candidiasis. The lesions are significantly more extensive when mucositis is induced. This model has been widely used and adapted in recent studies to evaluate new therapeutic approaches (Katagiri et al. 2018; Sampaio et al. 2024; Ninomiya et al. 2023).

Another rodent model of oral candidiasis is denture stomatitis in rats, which uses acrylic palatal devices to induce oral infection, associated with biofilm formation on these devices and/or inoculation of *C. albicans* on the palate before fixation of the device, since the denture itself is considered a predisposing factor for denture stomatitis (Aoun and Cassia 2016). This model has been studied since the pioneering work of Olsen and Bondevik (1978), who observed erythematous lesions in the mucosa beneath the device, characterizing inflammation. One study evaluated histological parameters, such as epithelial thickness and mitotic activity, and associated these findings with the inflammatory response triggered by *Candida* in tissues, suggesting that increased epithelial cell mitotic activity is linked to the observed inflammatory process (Shakir et al. 1986).

Some authors used immunosuppressants and maintained the devices for up to 72 h, a much shorter period than in other studies, which left the devices in place for up to 4 weeks. In histological analysis, hyphal invasion of the tissue was found, along with inflammation characterized by the infiltration of polymorphonuclear cells into the keratinized and deeper layers of the epithelium (Nett et al. 2010). Other authors, even without the use of immunosuppressants, were able to observe inflammatory signs similar to denture stomatitis and, histologically, tissue invasion and an increased inflammatory infiltrate in the connective tissue (Tobouti et al. 2016). In contrast, studies conducted with immunocompetent animals demonstrated that it was not possible to induce denture stomatitis due to the absence of erythematous lesions, neither fungal presence nor inflammatory infiltrates were observed histologically, with only epithelial thinning and loss of the papillary pattern between the epithelium and connective tissue being verified (Sakima et al. 2022). However, in a recent experimental model using immunocompetent rats, prior administration of antibiotics (tetracycline and amoxicillin) in the drinking water enabled the induction of evident clinical lesions on the palate. Histologically, microabscesses, disorganization of the basal layer, intracellular edema, and tissue invasion by yeasts were observed, in addition to inflammatory infiltrate in the connective tissue, findings that were more evident at the time of device removal. Although no significant differences were identified in the histometric parameters between the antibiotics tested, tetracycline was associated with greater local inflammatory activity (Moraes et al. 2022).

These animal models have also been used to evaluate innovative therapeutic strategies. Given the increasing challenge of fungal resistance, the search for alternative therapies has intensified. In this context, antimicrobial photodynamic therapy (aPDT) has shown promise in reducing the viability of *C. albicans* biofilms in immunosuppressed mice (Mima et al. 2010; Dovigo et al. 2013; Carmello et al. 2016; Jordão et al. 2023). Other approaches, such as the use of probiotics, as well as natural and synthetic compounds, have also proven to be effective in reducing *C. albicans* colonization in the oral mucosa (Ryan et al. 2014; de Campos Rasteiro et al. 2014; Matsubara et al. 2012).

## 3 Invasive Candidiasis

The terms “candidemia” and “invasive candidiasis” may cause confusion regarding their actual meaning, but it is important to clarify that they are not synonymous. While candidemia is defined as a severe fungal infection characterized by the presence of *Candida* in the bloodstream, invasive candidiasis is a broader term that encompasses both candidemia and other forms of *Candida* infection in deep organs and tissues. According to the guidelines of the Infectious Diseases Society of America (Pappas et al. 2016), there are three presentations of invasive candidiasis: candidemia without deep‐seated tissue involvement; candidemia associated with organ infections; and deep‐seated infections without fungemia. Therefore, candidemia is considered one of the possible manifestations of invasive candidiasis and, in many cases, is the main pathway through which the fungus spreads throughout the body (Clancy and Nguyen 2013; Pappas et al. 2016; Mora Carpio and Climaco 2025; Mallick et al. 2025).

Here, we summarize some information about invasive candidiasis, especially candidemia, to focus on the possibility of oral candidiasis disseminates through the body. Other types of invasive candidiasis, deep‐seated infections or systemic candidiasis, such as endophthalmitis, *Candida* endocarditis, meningitis due to the involvement of the central nervous system, disseminated candidiasis in patients with neutropenia, hepatosplenic candidiasis, or chronic disseminated candidiasis are very well described in other reviews (Pappas et al. 2016; Lass‐Flörl et al. 2024; Cornely et al. 2025), as well the host immunologic response.

According to the international cohort study of adult patients performed between 2019 and 2021 (Tabah et al. 2023), fungal infections accounted for 7.9% of hospital‐acquired bloodstream infections, second to those caused by Gram‐negative and Gram‐positive bacterial species. Among these fungal infections, 39.6% are caused by *C. albicans*, 57.8% by NACRY, and 6% by other fungi. The annual incidence of candidemia is 8.05 per 100,000 population, with a mortality rate ranging from 35% for treated patients to 90% for untreated patients; approximately 65% of deaths are attributable to *Candida* infection (Denning 2024). Invasive candidiasis may occur with negative blood culture, and its estimated annual incidence (12.1 per 100,000 population) is higher than that of candidemia, as are its associated mortality rates.

Blood culture testing used to diagnose candidemia has limited sensitivity and specificity and is influenced by some factors, including the volume of blood collected and prior antifungal prophylaxis or therapy. The lack of a standard diagnosis test for invasive candidiasis hinders accurate estimation of its mortality rate; therefore, mortality estimates for candidemia are often used as a proxy for invasive candidiasis (Denning 2024). Elderly population and infants exhibit a higher incidence of invasive candidiasis due to comorbidities in the former and the incomplete development of the immune system and epithelial barriers in the latter, especially neonates and preterm births (Lass‐Flörl et al. 2024; Bays et al. 2024; Warris and Ferreras‐Antolin 2026). Male sex and black people are more affected, probably because of socioeconomic reasons and access to the healthcare system (Bays et al. 2024; Lass‐Flörl et al. 2024).

The risk factors for candidemia, as well as some of the risk factors for oral candidiasis, include immunocompromised patients, individuals who use broad‐spectrum antibiotics, corticosteroids, radiotherapy, and chemotherapy (L. P. Samaranayake et al. 2009; Worthington et al. 2010; Telles et al. 2017). Increased risk for candidemia are those who underwent solid organ or hematopoietic stem cell transplantation, neutropenia (neutrophils < 500/μL), and critically ill individuals, such as those with COVID‐19 (Cesaro et al. 2018; Seagle et al. 2022; Lass‐Flörl et al. 2024). The development of invasive candidiasis is commonly associated with immunosuppression, rupture of epithelial barrier integrity by medical devices or mucosal inflammation and increased *Candida* load caused by antibiotioc therapy (Lass‐Flörl et al. 2024). Breaches in skin or mucosa caused by hemodialysis, urinary or intravascular catheters, indwelling prosthetic devices, ventricular drains and shunts, ureteral stents, prosthetic heart valves, endotracheal tubes, and so forth increase the probability of invasive candidiasis (Lass‐Flörl et al. 2024), since *Candida* forms biofilm inside these devices and shows reduced susceptibility to antifungal agents (Kojic and Darouiche 2004). Therefore, the management of invasive candidiasis involves the removal of the infected device coupled with an appropriate antifungal treatment (Kojic and Darouiche 2004; Lass‐Flörl et al. 2024).

The high mortality rate in patients with candidemia is strongly related to delayed antifungal therapy (Morrell et al. 2005; Playford et al. 2009), which is attributed to nonspecific clinical manifestations of candidemia similar to systemic infections caused by bacteria. Without a precise diagnosis, patients are first treated with antibiotics without success and antifungals are administered only after the lack of response to antibiotic therapy and persistence of clinical symptoms (Mouratidou et al. 2025). In this context, a retrospective cohort study of patients with candidemia observed that the timing of fluconazole therapy initiation in hospitalized patients with candidemia had a significant impact on mortality (Garey et al. 2006). These findings highlight the importance of rapid diagnostic testing to enable the earliest possible initiation of treatment. Therefore, rapid identification of candidemia is crucial, as delays in treatment can increase mortality, especially in the first days after infection. Blood culture is the standard detection practice, but it has low sensitivity and/or specificity and requires large volumes of blood. In addition, the growth and identification process of *Candida* spp. in culture may take 24–48 h (Chen et al. 2021).

Diagnosing invasive candidiasis is challenging because of the lack of specific signs and symptoms, which often resemble those of bacteremia and sepsis. Clinical manifestation are frequently nonspecific, difficult to identify in newborns, and may include persistent fever and chills, particularly during antibiotic treatment; skin lesions, such as bumps, pustules, or nodules; hypotension; multiorgan dysfunction; abdominal pain, which may indicate dissemination to liver and kidney; visual disturbance, including cotton like spots or blurry vision when infection affect the eyes and leads to ophtalmitis; muscle aches accompanied by generalized weakness and discomfort; and respiratory distress (Edman‐Wallér et al. 2016; Lass‐Flörl et al. 2024). Invasive candidiasis may also progress to septic shock, potentially resulting in renal and hepatic failure (Delaloye and Calandra 2014). Moreover, the absence of a diagnostic test with high sensitivity and specificity remains a major limitation, hindering a rapid diagnosis of invasive candidiasis and highlighting the need for further advances in this area (Delaloye and Calandra 2014; Lass‐Flörl et al. 2024).

### 3.1 Candidemia and NACRY

Currently, candidemia represents one of the most significant nosocomial infections associated with high mortality rates (Ben‐Ami 2018). This condition affects individuals with impaired immune function, such as oncology patients, transplant recipients, or individuals with Severe Combined Immunodeficiency, in whom immune deficiency or absence favors infection progression and, in some cases, leads to fatal outcomes (Vitiello et al. 2023).

Although *C. albicans* remains the main etiological agent, invasive candidiasis is also attributed to NACRY species, including *N. glabratus*, more frequently reported in the United States and Europe; *C. parapsilosis* and *C. tropicalis*, commonly observed in Latin America, Southern Europe, India, and Pakistan; *P. kudriavzevii*, prevalent in Europe, North America, and lowest Asia; and, more recently, *C. auris*, which has emerged as a global public health concern due to its multidrug resistance (Otto and Babady 2023; Lass‐Flörl et al. 2024; Pappas et al. 2018). Consequently, several studies have sought to correlate specific species with distinct risk groups. Evidence indicates that *N. glabratus* shows higher prevalence among elderly patients, those with diabetes, solid organ or hematopoietic stem cell transplants, renal failure, stroke, solid tumors, and hematological malignancies (Pappas et al. 2018; Sobel 2006). *C. parapsilosis* is commonly associated with infections in neonates, patients with cardiac valve disease or undergoing catheterization, those with solid tumors or hematological malignancies, individuals who have recently undergone surgery, and patients with peripheral or long‐term central venous catheters (CVCs) (Bonassoli et al. 2005; Sobel 2006; Silva et al. 2012; McCarty and Pappas 2016; Otto and Babady 2023; Pappas et al. 2018).

*C. tropicalis* is more frequently isolated in patients with hematological malignancies, leukemia, solid tumors, stem cell transplantation, severe neutropenia, or those who have undergone surgical procedures (Sobel 2006; Pfaller et al. 2012). Reports of *P. kudriavzevii* indicate its occurrence in patients with hematological malignancies, prolonged exposure to azole antifungals, neutropenia, and stem cell transplant recipients (Pfaller et al. 2012; Jamiu et al. 2021). Less common than the aforementioned species, *C. dubliniensis* has been reported in patients undergoing dialysis, those with Acquired Immunodeficiency Syndrome (AIDS) or HIV infection, and in individuals with hematological malignancies (Pfaller et al. 2012). *M. guilliermondii* is associated with patients with prior frequent antifungal use, severe neutropenia, and cancer (Otto and Babady 2023; Pfaller et al. 2012). Finally, studies have described *C. lusitaniae* infections in patients with solid tumors, surgical procedures, and cancer (Pfaller et al. 2012; Mendoza‐Reyes et al. 2022).

The treatment of candidemia involves various antifungal classes, with triazoles such as fluconazole widely used for less severe cases. Polyenes, particularly amphotericin B (Ben‐Ami and Kontoyiannis 2021), and echinocandins represent relevant therapeutic options, with the latter considered the first‐line therapy for critically ill, neutropenic, or advanced candidemia patients (Pappas et al. 2016). In contrast to bacterial infections, for which a broad range of antibiotics is available, antifungal therapeutic options remain limited, posing a significant public health challenge (Fisher et al. 2022). Despite the therapeutic efficacy of these agents, antifungal resistance has been increasingly reported in both *C. albicans* and NACRY species (Ben‐Ami and Kontoyiannis 2021; Lass‐Flörl et al. 2024).

Resistance may result from indiscriminate use of antifungal agents, often associated with self‐medication and the lack of strict prescription protocols, and can be classified as intrinsic (primary) or acquired (secondary) (Ben‐Ami and Kontoyiannis 2021). In addition, spontaneous or acquired genetic mutations and rearrangements have facilitated microbial adaptation to available therapies (Vitiello et al. 2023). In NACRY species, factors such as adaptive mutations, expression of virulence factors, and the degree of host immunosuppression influence variability in drug response (Deorukhkar et al. 2014). For instance, *C. albicans* and *C. tropicalis* exhibit high virulence but do not necessarily display universal antifungal resistance. In contrast, *P. kudriavzevii* exhibits intrinsic resistance to fluconazole, highlighting that virulence and resistance are not directly proportional attributes (Deorukhkar et al. 2014; Pappas et al. 2018).

Among the species with the highest fluconazole resistance are *N. glabrata*, *C. parapsilosis*, and *C. auris*, the latter being not only multidrug‐resistant but also described as pan‐resistant (Du et al. 2020). *N. glabratus* also demonstrates resistance to echinocandins (Healey et al. 2016), whereas *P. kudriavzevii* shows reduced susceptibility to amphotericin B (Pappas et al. 2016) and resistance to fluconazole (Lockhart et al. 2012). Meanwhile, *C. lusitaniae* exhibits resistance to amphotericin B and variable susceptibility to different antifungal classes (Rojas et al. 2024). The clinical relevance of fungal infections has increased globally, prompting the WHO to develop its first list of priority fungal pathogens, classifying *C. auris and C. albicans* as critical priority; *N. glabrata*, *C. tropicalis*, and *C. parapsilosis* as high priority; and *P. kudriavzevii* as medium priority (Vitiello et al. 2023). This scenario underscores the urgent need for further investigation of these pathogens, both from an epidemiological perspective and to develop new therapeutic strategies (Du et al. 2020).

*C. auris*, first described in Japan in 2009 from the ear secretion of a hospitalized patient, has emerged as a multidrug‐resistant pathogen, representing a significant public health concern and attracting increasing global attention (Du et al. 2020). In a European cohort study, this species was among the six most frequently isolated (Hoenigl et al. 2023). It is a haploid fungus, often associated with hospital environments, invasive infections, and nosocomial outbreaks (Du et al. 2020). Its remarkable ability to colonize and persist on the skin and environmental surfaces for extended periods distinguishes it from other species of NACRY (Eix and Nett 2025). Major risk factors associated with *C. auris* infections include prolonged use of catheters and medical devices, antibiotic prophylaxis, prior surgical procedures, extended stays in ICUs, and underlying clinical conditions (Sayeed et al. 2020). Furthermore, these infections have been reported in association with comorbidities, such as diabetes, sepsis, chronic kidney disease, and pneumonia, emphasizing the importance of host clinical status in disease progression (Chowdhary et al. 2013; Eix and Nett 2025).

Fluconazole‐resistant *C. parapsilosis* has emerged as a global threat due to its association with hospital outbreaks (Lass‐Flörl et al. 2024), its persistence despite infection control measures, and its potential for widespread dissemination, underscoring the need for enhanced surveillance (Fekkar et al. 2023). Moreover, the emergence of multidrug‐resistant and echinocandin‐tolerant *C. parapsilosis* strains highlights the importance of further studies investigating the virulence and molecular features of this species. Such efforts may facilitate monitoring of circulating genotypes and support the development of effective therapies and infection control strategies to contain these clonal outbreaks (Daneshnia et al. 2023).

### 3.2 Animal Model of Candidemia

Murine models of candidemia have played a fundamental role in improving our understanding of how the disease begins and develops. In addition, they allow diagnostic and therapeutic strategies to be tested with greater precision, contributing to advances in both basic knowledge and clinical practice (Jungnickel and Jacobsen 2022).

Historically, the most common practice has been intravenous inoculation of *Candida*, which consists of injecting fungal cells directly into the bloodstream of mice, thereby allowing researchers to follow how the fungus disseminates throughout the organism and how the immune system responds to it (MacCallum and Odds 2005). This type of model has been essential for identifying the main target organs, such as the kidneys, liver, intestine, and spleen, and has also paved the way for evaluating the efficacy of antifungal therapies and mechanisms of virulence (Szabo and MacCallum 2011). In addition to this method, another relevant approach is the gastrointestinal colonization and dissemination model, in which *C. albicans* is administered by oral gavage or in drinking water. For intestinal colonization to be effective, however, the animals must be immunosuppressed (Koh 2013).

Although this approach has been used for a long time, murine models continue to provide relevant information. The possibility of standardizing experiments enables researchers to compare different strains, study the efficacy of new antifungal agents, and understand the interaction between *Candida* and host defenses. Therefore, despite the limitations inherent to any experimental model, animal studies remain an important tool for clarifying the processes involved in candidemia (Jungnickel and Jacobsen 2022). More recently, invertebrate models have been encouraged to replace vertebrate animals, thus nematodes and insects, such as the worm *Caenorhabditis elegans*, the vinegar fly *Drosophyla melanogaster*, and the greater wax moth *Galleria mellonella*, have been used to investigate fungal infections, since many characteristics of the innate immune system are conserved between mammals and invertebrates (Arvanitis et al. 2013; Mylonakis et al. 2007; Vega‐Chacón et al. 2021).

## 4 May Oral Candidiasis Spread to Systemic Infection?

Since mucosal inflammation is a risk factor for invasive candidiasis (Lass‐Flörl et al. 2024), oral candidiasis is a potential source of *Candida* dissemination. It is well documented that *C. albicans* is able to colonize the gastrointestinal tract, adapt to the gut environment and interact with the gut microbiome. Under circumstances that disrupt the host homeostasis, such as immunosuppression, antibiotic therapy, and epithelial damage, the yeast‐to‐hyphae transition promotes invasion into the gut epithelium and fungal cells may reach the bloodstream and disseminate to distant organs, causing invasive candidiasis (Koh 2013; Basmaciyan et al. 2019; Sprague et al. 2022). On the other hand, few studies have reported the evidence that oral *Candida* infection disseminates in systemic infection, which has been evaluated in murine models (Clemons et al. 2006; Mosci et al. 2014; Katagiri et al. 2018; Kobayashi‐Sakamoto et al. 2018; Ninomiya et al. 2023; Veerapandian et al. 2025). These investigations have demonstrated that the dissemination of *Candida* occurs mainly via the gastrointestinal tract (Kobayashi‐Sakamoto et al. 2018; Veerapandian et al. 2025).

A study conducted in China detected the presence of *C. albicans* strains isolated from different body sites of patients admitted to the ICU and, as a result, identified the same genotypes in strains isolated from the oral cavity and blood samples of some of these individuals (Tan et al. 2021). In murine models, there are few investigations that demonstrate the relationship between oral candidiasis and invasive candidiasis, although some plausible considerations can be made. One of the earliest murine studies demonstrating the progression from mucosal candidiasis to systemic dissemination was described by Clemons et al. (2006). In this orogastrointestinal model, immunosuppression was induced using 5‐fluorouracil prior to oral exposure to *C. albicans*, which was administered in the drinking water, allowing natural ingestion and colonization of the oral cavity and gastrointestinal tract. This model resulted in mucosal infection followed by dissemination to visceral organs, with fungal burdens detected in the liver and kidneys within days after infection (Clemons et al. 2006).

Additional experimental evidence supporting the progression from mucosal candidiasis to systemic infection has been demonstrated in a murine model lacking interleukin 17A (IL‐17A, a pro‐inflammatory cytokine produced mainly by T helper 17 lymphocytes that acts in the immune response against fungal infection). In this model, mice treated with corticosteroids were orally infected with *C. albicans*, and the progression of infection was monitored using real‐time bioluminescence imaging. In the absence of IL‐17A, fungal infection extended from the oral cavity to the esophagus and stomach and caused severe damage to the intestinal mucosa, favoring fungal translocation. Dissemination to visceral organs, including the liver and kidneys, was observed, where fungal abscesses were detected. Although neutrophils were recruited to infected tissues, their candidacidal activity was impaired, highlighting the critical role of IL‐17A in preventing the transition from mucosal candidiasis to systemic infection (Mosci et al. 2014).

An experimental study in mice evaluated the association between mucositis and fungemia. In groups in which mucositis was induced, *C. albicans hyphae* were observed infiltrating the subepithelium and blood vessels, leading to fungemia, with a higher incidence in the chemotherapy group. These findings indicate that mucositis facilitates the dissemination of *Candida* into the bloodstream (Katagiri et al. 2018).

Another study induced oral candidiasis in immunosuppressed mice to establish an experimental model of gastrointestinal and disseminated candidiasis (Kobayashi‐Sakamoto et al. 2018). To achieve effective immunosuppression, tetracycline was administered in drinking water to alter the oral microbiota, and prednisolone was used as a systemic immunosuppressive agent prior to oral inoculation with *C. albicans*. Two study groups were evaluated: mice that received prednisolone and a control group that received physiological saline as a placebo. Prednisolone not only increased the fungal burden in gastrointestinal organs (tongue, stomach, small intestine) but also facilitated dissemination to internal organs such as the spleen and liver within just 3 days after infection. In addition, histological analysis revealed hyphal infiltration in organs such as the tongue and stomach, especially in mice treated with the immunosuppressive agent. These findings suggest that the initial local infection can rapidly progress to a systemic infection (Kobayashi‐Sakamoto et al. 2018).

Recent evidence from the same research group showed that antifungal treatments, both oral topical miconazole and systemic fluconazole, prevented systemic fungemia triggered by oral candidiasis in mice (Ninomiya et al. 2023). In this model, the disease was induced via mucositis, with animals receiving systemic chemotherapy and oral inoculation with *C. albicans*. As also described by Katagiri et al. (2018), mucositis was accompanied by oral ulceration, creating favorable conditions for the fungus to reach the bloodstream. The presence of *Candida* in the blood was confirmed by plating, followed by PCR identification of the grown colonies. These findings reinforce the hypothesis that oral candidiasis can, under certain circumstances, serve as a gateway to candidemia.

Dissemination can occur even without the use of antibiotics, when there is immunosuppression. In a murine model, oral candidiasis induced solely by cortisone progressed to mucosal invasion, reached the gastrointestinal tract, and spread to the liver. Furthermore, a profound alteration in the microbiome was observed, marked by the loss of beneficial bacteria and the growth of pathogenic bacterial species. These findings indicated that isolated immunosuppression, associated with dysbiosis, already creates conditions for oral infection to develop into a systemic condition (Veerapandian et al. 2025).

In summary, to the best of our knowledge, only six studies have demonstrated that oral candidiasis disseminated into invasive candidiasis in immunosuppressive mice submitted to corticosteroid or chemotherapy (Clemons et al. 2006; Mosci et al. 2014; Katagiri et al. 2018; Kobayashi‐Sakamoto et al. 2018; Ninomiya et al. 2023; Veerapandian et al. 2025), the fungal translocation took place through gastrointestinal tract (Clemons et al. 2006; Kobayashi‐Sakamoto et al. 2018; Veerapandian et al. 2025) and topical and systemic antifungal therapy prevented the dissemination (Ninomiya et al. 2023). Further investigations are warranted to better understand the mechanisms of dissemination, the host defense responses activated during fungal spread and the development of effective therapies to prevent dissemination without inducing antifungal resistance.

## 5 Treatments

For the treatment of infections caused by *Candida* spp. and other fungi, antifungal agents are used. Unlike antibiotics, only a few classes of antifungal agents are available, and developing new antifungals without toxicity to host tissues is challenging due to similarities between mammalian and fungal cells. The main classes of antifungal agents are polyenes, azoles, and echinocandins (Ghannoum and Rice 1999; Aliaghazadeh et al. 2025).

Polyenes were the first class of antifungal agents and act by altering the permeability of the fungal cell membrane, resulting in a fungicidal effect. This effect occurs because polyenes bind to ergosterol in the cell membrane, altering its permeability, a mechanism that in some studies has been referred to as the “ergosterol sponge.” This class includes nystatin and amphotericin B (Puumala et al. 2024; Ghannoum and Rice 1999). Although amphotericin B showed side effects, such as nephrotoxicity, liposomal formulations improved its toxicity and tolerability (Hamill 2013).

Azoles were the second antifungal class developed and considered a milestone in antifungal treatment, due to their efficacy and safety, especially with the broad‐spectrum systemic fluconazole during the HIV pandemic. Azoles exert a fungistatic effect by inhibiting the enzyme lanosterol 14‐α‐demethylase (encoded by the *ERG11* gene). This inhibition leads to the accumulation of toxic sterols and, consequently, blocks the synthesis of ergosterol, an important lipid component of the fungal cell membrane. Azole antifungals are subdivided into two groups: imidazoles (such as ketoconazole and miconazole) and triazoles (such as fluconazole and itraconazole). The structural difference between them lies in the number of nitrogen atoms: imidazoles contain two, whereas triazoles have three (Puumala et al. 2024; Ghannoum and Rice 1999). However, due to their fungistatic action, resistance to azoles is very common and poses a challenge to the health field (Puumala et al. 2024).

Echinocandins represent a more modern class of antifungals compared with the classes mentioned above, consisting of semisynthetic drugs derived from natural substances. Their mechanism of action involves blocking the enzyme 1,3‐β‐d‐glucan synthase (encoded by the *FKS1* gene), which is essential for the synthesis of structural components of the fungal cell wall. Because they are systemically administered by injection, they are reserved only for severe cases, especially systemic infections. This group includes caspofungin, micafungin, anidulafungin, and, more recently, rezafungin (Ghannoum and Rice 1999; Garcia‐Effron 2020; Puumala et al. 2024).

There is also the antifungal class known as pyrimidine analogs, represented by 5‐flucytosine (5‐FC). Although it is not indicated for the treatment of oral candidiasis, it deserves mention due to its relevance in other fungal infections. Discovered in 1970, 5‐FC is converted to 5‐fluorouracil (5‐FU) within the fungal cell, leading to inhibition of DNA and RNA synthesis and thereby inhibiting fungal growth. Because resistance develops rapidly, it is not used as monotherapy but in combination with amphotericin B. In this association, it is indicated for the treatment of fungal meningitis caused by *Cryptococcus* spp. and *Candida* spp., as well as endocarditis, endophthalmitis, symptomatic urinary tract infections caused by azole‐resistant species, neonatal candidiasis (due to the high frequency of meningeal involvement), and also for infections caused by dematiaceous fungi, such as phaeohyphomycosis and chromoblastomycosis (Sigera and Denning 2023).

### 5.1 Treatment of Oral Candidiasis

Oral candidiasis can be treated with two modalities: topical and systemic medications. Topical drugs are usually used in the early stages of oral candidiasis, such as nystatin and miconazole, and are the first‐line treatment. These drugs have been used for a long time and have proven effective in relieving the signs and symptoms of this oral infection. However, studies show that when treatment does not completely eliminate the microorganisms, recurrence may occur (Perezous et al. 2005).

In such situations, systemic medications are necessary. This modality is mainly indicated for immunosuppressed patients or those who have had disease recurrences. Fluconazole, itraconazole, and amphotericin B are drugs that can be used to combat *Candida* species (Perezous et al. 2005; Garcia‐Cuesta et al. 2014). However, amphotericin B can cause undesirable effects, as it is considered hepatotoxic and nephrotoxic (Lombardi and Budtz‐Jorgensen 1993). To overcome this cytotoxicity, liposomal amphotericin B was developed, in which the drug is encapsulated in liposomes. This modification allows for more controlled release, reducing systemic toxicity and improving treatment tolerability (Hamill 2013).

Topical or systemic antifungal therapy faces significant obstacles due to the emergence of resistant phenotypes in species of the genus *Candida*. Part of this resistance is associated with the fungistatic nature of azole antifungals, which are not capable of completely eliminating the microorganisms (Cartledge et al. 1997; Bhattacharya et al. 2020). At the molecular level, resistance involves genetic and regulatory alterations, such as the overexpression of efflux pumps and mutations in genes related to ergosterol biosynthesis (*ERG11*) (Hu et al. 2023; Chitharagi et al. 2026). Furthermore, modifications in transcription factors (such as *TAC1*) (Fan et al. 2023) and mutations in other genes, such as *MRR1* (Branco et al. 2022), have been associated with azole resistance. In summary, prolonged use of antifungals may lead to the selection of fungal populations with adaptive phenotypes that reduce therapeutic efficacy (White et al. 1998; Huang et al. 2025). For this reason, there is a need to investigate and develop new alternative therapies as treatment options.

aPDT has been increasingly highlighted and involves the combination of a photosensitizing agent with a light source of appropriate wavelength in the presence of oxygen, which generates reactive oxygen species that are toxic to the target cell (Baltazar et al. 2015). Other modalities, such as antimicrobial peptides, natural compounds, new triazoles, and synergism between alternative and conventional agents, have been investigated (de Oliveira Santos et al. 2018).

### 5.2 Treatment of Candidemia

Antifungal therapy for *Candida* infections should be guided by species identification. Although *C. albicans* generally remains susceptible to most antifungal classes, including azoles, echinocandins and amphotericin B, several NACRY species exhibit intrinsic or acquired resistance that may limit therapeutic options. For example, *P. kudriavzevii* is intrinsically resistant to fluconazole, *C. lusitaniae* may display resistance to amphotericin B, and *C. parapsilosis* frequently shows reduced susceptibility to echinocandins and is resistant to fluconazole. In addition, species such as *N. glabratus* and *C. auris* are often associated with reduced susceptibility to multiple antifungal classes and may develop multidrug resistance during treatment. Consequently, accurate species identification and antifungal susceptibility testing are essential to guide appropriate therapy and optimize clinical outcomes (Lass‐Flörl et al. 2024).

For the treatment of candidemia, the first‐line therapy of choice is echinocandins, as they offer good safety, few drug interactions, and efficacy against various *Candida* species. If this class is not available or in cases of resistance, the second‐line therapy includes liposomal amphotericin B, fluconazole, or voriconazole (Cornely et al. 2025). Table 4 summarizes the current treatment of candidemia.

**Table 4:** Recommended antifungal therapy for candidemia (adapted from Cornely et al. 2025).

Category	Antifungal drugs	Recommended dose
First‐line treatment	Anidulafungin	200 mg once a day on day 1 and 100 mg daily from day 2
	Caspofungin	70 mg once a day on day 1; 50 mg daily from day 2
	Micafungin	100 mg daily
	Rezafungin	400 mg once a week in week; 200 mg weekly from week 2
Second‐line treatment	Liposomal amphotericin B	3 mg/kg daily
	Voriconazole	6 mg/kg twice a day on day 1; 4 mg/kg twice a day from day 2
	Fluconazole	400–800 mg daily

In addition to the appropriate choice of antifungal, the clinical management of candidemia also requires identification and control of the infectious focus, especially when associated with intravascular devices or intra‐abdominal infections. In such cases, removal of the CVC, whenever possible, drainage of abscesses, and early initiation of antifungal treatment are indispensable (Hashemi Fesharaki et al. 2018).

The recommendation for the treatment of candidemia, when there are no signs that the infection has spread to internal organs, is to maintain antifungal therapy for 14 days from the first negative blood culture that remains consistently negative. In other words, the duration of therapy does not begin when the antifungal is initiated, but rather from the moment the fungus is no longer detected in the bloodstream, ensuring that treatment is sufficient to prevent relapses (Cornely et al. 2025).

In cases involving internal organs, such as the central nervous system, endocarditis, or ocular involvement, the treatment of invasive candidiasis requires specific approaches due to the clinical complexity of these manifestations. Other less common forms, such as abdominal candidiasis, chronic disseminated candidiasis, osteoarticular infections, urinary tract infections, *Candida* pneumonia, and thoracic empyema, also present therapeutic particularities that must be considered. Detailed information on the management of these presentations is available in the Global Guideline for the Diagnosis and Management of Candidiasis (Cornely et al. 2025).

## 6 Conclusion

Oral candidiasis remains the most common fungal infection of the oral cavity, but its importance extends beyond local manifestations. It has been suggested that oral candidiasis may act as a reservoir for *Candida*, potentially contributing to the development of more severe forms, such as candidemia, particularly in immunocompromised individuals. Only one review is available about the relationship between oral candidiasis and candidemia in patients hospitalized in ICU, highlighting the relevance of oral hygiene care in controlling fungal overgrowth and preventing morbidity and mortality (Ferreira et al. 2023). The increasing involvement of NACRY, along with growing antifungal resistance and global outbreaks, underscores the urgency of developing new therapeutic and preventive strategies. Moreover, the interactions between *Candida* spp. and the oral microbiome remain poorly understood, offering avenues for future research. Thus, clarifying whether oral infection can promote systemic dissemination is essential to improve early diagnosis and to guide control measures that may reduce the clinical and hospital impacts of invasive candidiasis.

## Author Contributions

**Julia Robledo Jerez:** investigation (oral candidiasis, candidemia, animal model, and treatment), writing, image acquisition, formatting. **Marcella Vieira Ambrosio:** investigation (oral microbiome and non‐*albicans Candida* and related yeasts), writing, formatting. **Marcela Eduarda Olegario Fernandes:** investigation (non‐*albicans Candida* and related yeasts), writing. **Ewerton Garcia de Oliveira Mima:** supervision, conceptualization, writing, reviewing, and editing.

## Ethics Statement

The authors have nothing to report.

## Conflicts of Interest

The authors declare no conflicts of interest.

## Data Availability

The authors have nothing to report.
